# Deaths Associated with Influenza Pandemic of 1918–19, Japan

**DOI:** 10.3201/eid1904.120103

**Published:** 2013-04

**Authors:** Siddharth Chandra

**Affiliations:** Michigan State University, East Lansing, Michigan, USA

**Keywords:** influenza viruses, pandemic, Japan, 1918, 1918–19, deaths, viruses

## Abstract

Current estimates of deaths from the influenza pandemic of 1918–19 in Japan are based on vital records and range from 257,000 to 481,000. The resulting crude death rate range of 0.47%–0.88% is considerably lower than parallel and conservative worldwide estimates of 1.66%–2.77%. Because the accuracy of vital registration records for early 20th century Asia is questionable, to calculate the percentage of the population who died from the pandemic, we used alternative prefecture-level population count data for Japan in combination with estimation methods for panel data that were not available to earlier demographers. Our population loss estimates of 1.97–2.02 million are appreciably higher than the standing estimates, and they yield a crude rate of population loss of 3.62%–3.71%. This rate resolves a major puzzle about the pandemic by indicating that the experience of Japan was similar to that of other parts of Asia.

The influenza pandemic of 1918–19 caused unprecedented devastation (*1*); worldwide, it is estimated to have taken 25–100 million lives (*2*,*3*), exceeding the combined death toll of both world wars. One of the strangest aspects of the currently held wisdom about the pandemic is the curiously low death rate attributed to Japan compared with other countries in Asia. Official records for Japan put the death toll at 257,363 persons (*4*), resulting in a crude influenza-attributable death rate of 0.47%. Patterson and Pyle (*2*) reported 350,000 deaths, and Johnson and Mueller (*3*) cited a figure from Palmer and Rice (*5*) of 388,000 deaths. Given Japan’s population of >54 million at the time (*6*), the influenza-attributable mortality rates (0.64%–0.71%) are remarkably low by Asian standards, although they are similar to the rates calculated for the United States, Canada, and western Europe (0.65%, 0.61%, and ≈0.48%, respectively) (*3*). Patterson and Pyle’s (*2*) conservative estimate of a global rate of 1.66% and Johnson and Mueller’s (*3*) substantial upward revision of that percentage to 2.77% suggest that the estimates for Japan, which are less than one quarter of the latter estimate, merit closer scrutiny. Although the epidemiologic approach used by Richard et al., which also uses death statistics reported by the Japanese health authorities, raises the estimate to 481,000 (or 0.88% of the population at the time) (*7*), even this estimate is extraordinarily lower than estimates from other parts of Asia.

As Taeuber argued in her classic book, *The Population of Japan*, Japan occupies a special place in demography (*8*). Worldwide it remains one of the largest economies (third in 2011) and one of the most populous countries (tenth in 2011). Yet, surprisingly, substantial knowledge gaps remain with regard to the influenza pandemic of 1918–19 in Japan, rendering it “a strangely neglected episode in modern Japanese history” (*4, p**. 389*). For example, a search of Taeuber’s work for the term “influenza” revealed only 1 mention of the influenza epidemic of 1918, in the context of speculation that it “may have led to reduced conceptions” (*8, p**. 233*).

The few scholars who have studied the influenza pandemic in Japan have approached it from 1 of 3 broad perspectives: historical, epidemiologic, or demographic. The historical approach is exemplified by the works of Palmer and Rice, which provide a qualitative contextualization of aspects of the pandemic and its management in Japan (*4*,*5*,*9*,*10*). A second line of research is epidemiologic, within which 2 broad goals are pursued. The first goal is to produce estimates of major epidemiologic characteristics of the virus (*11*–*13*), and the second goal is to produce epidemiology-based estimates of mortality rates from the pandemic (*7*). The demographic approach is exemplified by Morita, Okazaki, Taeuber, and Yasukawa and Hirooka (*8*,*14*–*16*). Although these studies emphasize broader patterns of population growth in Japan, a few address the question of death rates during the pandemic. For example, Yasukawa and Hirooka (*16*) relied directly on official death statistics, including those from the pandemic, to produce estimates of the population in early 20th-century Japan. Unfortunately, the quantitative literature seems to have more or less accepted the official vital statistics on disease-specific deaths, feeding them (and therefore their inaccuracies) into otherwise technically refined estimates of population and population growth.

A common characteristic of the above studies is their heavy reliance on official vital and health statistics of the time. Such data are widely recognized by demographers as being plagued by the often-severe problem of underreporting. Indeed, according to Johnson and Mueller, “it is generally accepted that recorded statistics of influenza morbidity and mortality are likely to be a significant understatement” (*3, p**. 108*). For India, Davis estimated that the “amount of underregistration certainly exceeds 30 per cent at all times, and is probably nearer 50 per cent” (*17, p**. 34*). For Indonesia, Gooszen advised that such data “should be regarded with a good deal of caution” (*18, p**. 32*), and Nitisastro opined that “for the system of registering deaths, the quality of the results was poor” (*19, p**. 101*). Japan is no exception to this pattern. According to Mosk, “we do not have a trustworthy picture of what happened to vital rates in the Tokugawa period…. The same can be said for the Meiji period” (*20, p**. 658*), and Taeuber’s assessment was that “the critical question is the accuracy of the records of vital events” (*8, p**. 50*). The uncharacteristically low estimates of deaths from influenza in Japan provide a strong rationale for cross-checking the findings in the manner of Davis’ classic study of India (*17*). 

We therefore used recently developed statistical methods to estimate the loss of population in Japan from the influenza pandemic of 1918–19. We adopted an approach that intentionally avoids heavy reliance on vital registration data and is based instead on population count data for Japan of that period. By applying data for multiple prefectures over time to prefecture-level population statistics, we estimated population loss from the pandemic to be the difference between expected population (using the prepandemic trajectory) and observed population (using the postpandemic trajectory) (*17*,*21*,*22*). The new estimates are appreciably higher than the earlier estimates, bringing Japan’s pandemic experience in line with that of other parts of Asia and resolving a major puzzle in the epidemiology of the 1918–19 pandemic.

## Methods

### Data-associated Issues

With regard to data, 3 issues should be considered. The first is the coverage of the population count data for Japan in the late 19th and early 20th centuries, described by Matsuda (*23*) and Taeuber (*8*). In 1871, the Imperial Japanese government passed a law, the *koseki-ho*, which required registration of households and persons in Japan. A major emphasis of the registrations was legal domicile, or *honseki* status. The first set of summations of these registers was made in 1898, after which they were computed every 5 years until 1918, for a total of 5 nationwide population counts derived directly from the registers (*8*,*24**–**26*). The number of persons who physically resided in different parts of Japan (de facto A-type population) was computed by adjusting the numbers of persons with *honseki* status downward to account for those who had *honseki* status but lived in other locations. These numbers were further adjusted to account for the discrepancy between numbers of registered persons who immigrated into the various prefectures, which always exceeded the numbers of persons who migrated out (de facto B-type population [*27*]). Over time, through a process of learning by doing (habituation), the registration data became reasonably accurate (*8*). 

For this study, we used data from the quinquennial (every 5 years) summations of 1898 and after. Given the de facto nature of the censuses of 1920 and after, we used the B-type population statistics for comparable data for 1918 and before (*6*). Because the population figures are based on repeated summations of records that were repeatedly updated, the count for a household was periodically revised upward or downward, and hitherto unreported births and deaths would have been more accurately captured by these revisions, even if they had not been reported in the annual vital registration records. Taeuber (*8*) provides evidence as follows: “Early publications of the Bureau of Statistics included a warning statement that the majority of the additions to the registers were the survivors of unrecorded births of earlier years,” and “Failures to report deaths during the earlier years are evident in the accumulations of the aged in the successive reports.” This phenomenon forms the basis for our reasoning that the quinquennial population count data are more accurate than the annual vital registration records.

The second data-associated issue is the change in the regime for population enumeration that began in 1920, when Japan conducted its first census of its de facto population. This census is widely regarded as having been accurate and yielded a population count of 55.96 million (*6*). After conducting the 1920 census, Japan conducted quinquennial censuses until the beginning of World War II. The problem with the timing of the change in the system is that quinquennial registration count totals are available up to 1918, and the quinquennial census counts started in 1920. Therefore, the break point in the system of population enumeration approximately coincides with the break point (1918) for which population loss is to be estimated. Any statistical estimation of change across such a break point must satisfactorily address discontinuity in the data collection system. Fortunately, earlier demographers and statisticians went to great lengths to splice the data across this break point, producing similar estimates. Taeuber (*8*), for example, demonstrated that a backward projection of population from 1920 through 1898 produces a 1898 population estimate that is remarkably similar to a forward projection of population from 1871 through 1898. The theme of splicing is also covered in the works of Morita, Okazaki, and Yasukawa and Hirooka (*14*–*16*). Ohbuchi (*28*) compared the estimates of these authors, all of which are within 2% of each other for 1915 and 1920, and concluded that the estimates of Yasukawa and Hirooka, which are based on a reverse survival method, are the most reliable. Although the procedure used by Yasukawa and Hirooka is generally robust, its adjustment for the influenza pandemic fails because the official influenza death statistics of ≈178,000 for 1918–1920 were taken at face value and incorporated into estimates of life expectancy at birth (*16*). Therefore, inaccuracies in the official vital statistics of the period flow directly into the estimates of Yasukawa and Hirooka. When selecting the data for the analysis, therefore, we started with the observations of Yasukawa and Hirooka (*16*), who stated that by 1900, the most widely used population estimates of demographers (*14*–*16*) tend to converge and are close to the official population statistics. Next, because the official statistics are the only ones that contain published data at the prefectural level (*6*) (Morita, Okazaki, and Yasukawa and Hirooka [*14*–*16*] focus on producing Japan-wide data), we used the official statistics pertaining to the quinquennial population count (1918 and before) and census years (1920 and after). 

The third data-related issue is the unreliability of the data before 1898 (*8*,*16*); therefore, we used the 1898 population count as the starting point in our analysis. To maintain balance of the dataset across the break point of 1918, we also limited our data to the censuses including and before 1935. The full dataset consists of observations for each of 47 prefectures for the population count for the years 1898, 1903, 1908, 1913, and 1918, and the census data for 1920, 1925, 1930, and 1935, for a total of 423 observations.

### Data Analyses

Although for decades scholars have been intrigued by the subject of low mortality rates from the pandemic in Japan, the currently circulating estimates were produced before the development and mainstreaming of panel data estimation methods. The studies described above based population estimates on annual or quinquennial observations for all of Japan and used datasets that were small in terms of numbers of observations. Given the existence of a panel of prefectural data on population for 47 prefectures and multiple time points straddling the pandemic years, more recently developed panel data methods can be used to estimate a standard population growth process that explicitly builds in a break point for the influenza pandemic (*21*,*22*). By treating these 47 prefectures of Japan as individual units, each with its own set of observations, the panel data method leverages the large amount of additional information available at the prefectural level to generate a more robust picture of population change and the effect of the influenza pandemic on that process. This method is also flexible enough, given the large sample size, to accommodate prefecture-specific variation. In this manner, the method enables estimation of prefecture-specific growth processes, each with a prefecture-specific estimate of population loss from the pandemic, while still leveraging the entire set of observations to create an aggregate estimate for Japan. This method is implemented by running a regression of the logarithm of population on a linear time trend while allowing for a 1-time (downward) shift in that time trend during 1918–19 to capture influenza-attributable population loss. Details of this method are provided in the Technical).

To examine the robustness of the estimates, we conducted a variety of sensitivity analyses. First, to control for the possible inaccuracy of the 1898 data and for the effects of outliers in time (the 1898 and 1935 data), we estimated models without these 2 time points. Second, we estimated models without the 4 prefectures that were most affected by the devastating Kanto earthquake of 1923: Chiba, Kanagawa, Shizuoka, and Tokyo. Third, because the 1918 population count was reported as of December of that year (i.e., the year of the pandemic), thereby introducing the possibility of contamination in the growth rate estimate for the prepandemic trajectory, we estimated models without the 1918 data. Finally, given the atypical population dynamic in Hokkaido, a frontier region in the early 20th century to which a large and prolonged wave of migration was in progress, we estimated models without data for Hokkaido. These sensitivity exercises yielded a total of 16 possible permutations of the model. Additional sensitivity analyses involved using the alternative A-type statistics (*6*) and dropping the data for 1898 (i.e., using 1903 as the earliest year) to account for the above-mentioned habituation process.

## Results

Tables 1 and 2 contain the parameter estimates for the 16 models. Without exception, the models show the significant negative effect of the influenza pandemic on Japan’s population (via the flu dummy described in the Technical Appendix); the calculated population loss ranged from 1.38 to 2.05 million persons. The range of estimated population growth rates across the models is 0.89%–1.13% per year, which is in line with the summary of estimates presented by Ohbuchi (*28*). In all but 1 model, there is no appreciable difference in the rates of population growth before and after the pandemic. As predicted, the inclusion of the population count data for 1918, which already reflect some but not all deaths from the pandemic, pulls the prepandemic population growth trajectory down (Figure), yielding substantially lower estimates of death and population loss than corresponding models that did not include those data (Tables 1, 2). For this reason, the models that exclude the 1918 data are preferred to the models that include the 1918 data.

**Table 1:** Population growth models and population loss estimates for Japan, 1903–1930 data*

Estimate	Model								
	1		2	3	4	5	6	7	8
Includes Kanto earthquake prefectures†	Yes		Yes	Yes	Yes	No	No	No	No
Includes Hokkaido outlier	Yes		Yes	No	No	Yes	Yes	No	No
Includes 1918 population count data	Yes		No	Yes	No	Yes	No	Yes	No
Intercept, γ_00_	13.6120‡		13.5957‡	13.6197‡	13.6040‡	13.5799‡	13.5623‡	13.5876‡	13.5706‡
	*0.0524*		*0.0530*	*0.0530*	*0.0535*	*0.0534*	*0.0537*	*0.0541*	*0.0543*
Time trend, γ_10_	0.0103‡		0.011‡	0.0095‡	0.0105‡	0.0098‡	0.0110‡	0.0089‡	0.0100‡
	*0.0012*		*0.0012*	*0.0009*	*0.0008*	*0.0012*	*0.0012*	*0.0009*	*0.0008*
Flu dummy, γ_20_	–0.0344‡		–0.0477‡	–0.0364‡	–0.0492‡	–0.0374‡	–0.0518‡	–0.0397‡	–0.0536‡
	*0.0055*		*0.0070*	*0.0052*	*0.0069*	*0.0055*	*0.0067*	*0.0051*	*0.0067*
Flu dummy × time trend, γ_30_	0.0006		–0.0004	0.0013§	0.0003	0.0002	–0.0010	0.0009	–0.0002
	*0.0009*		*0.0010*	*0.0006*	*0.0007*	*0.0009*	*0.0010*	*0.0006*	*0.0006*
No. observations	329		282	322	276	301	258	294	252
Hausman test statistic	<0.0001		<0.0001	<0.0001	<0.0001	<0.0001	<0.0001	<0.0001	<0.0001
	p>0.9999		p>0.9999	p>0.9999	p>0.9999	p>0.9999	p>0.9999	p>0.9999	p>0.9999
Breusch-Pagan test statistic	924.89		653.01	921.41	652.71	848.22	599.17	847.85	601.55
	p<0.0001		p<0.0001	p<0.0001	p<0.0001	p<0.0001	p<0.0001	p<0.0001	p<0.0001
Population change from influenza, millions	–1.38	–1.97		–1.50	–2.02	–1.48	–2.12	–1.61	–2.17
Population change from influenza, %	–2.53	–3.62		–2.87	–3.87	–3.15	–4.51	–3.59	–4.85
Population change, 1918–19, millions	–0.66	–1.21		–0.89	–1.38	–0.90	–1.49	–1.13	–1.65
Population change, 1918–19, %	–1.20	–2.19		–1.69	–2.60	–1.89	–3.13	–2.51	–3.65
Annual population growth rate to pandemic, %	1.03	1.13		0.95	1.05	0.98	1.10	0.89	1.00
Annual population growth rate after pandemic, %	1.09	1.09		1.08	1.08	1.00	1.00	0.98	0.98

*Italics indicate SE of the coefficient. †Chiba, Kanagawa, Shizuoka, Tokyo. ‡p<0.01. §p<0.05.

**Table 2:** Population growth models and population loss estimates for Japan, 1898–1935 data*

Estimate	Model							
	1	2	3	4	5	6	7	8
Includes Kanto earthquake prefectures†	Yes	Yes	Yes	Yes	No	No	No	No
Includes Hokkaido outlier	Yes	Yes	No	No	Yes	Yes	No	No
Includes 1918 population count data	Yes	No	Yes	No	Yes	No	Yes	No
Intercept, γ_00_	13.6053‡	13.5971‡	13.6137‡	13.6059‡	13.5755‡	13.5673‡	13.5839‡	13.5762‡
	*0.0523*	*0.0526*	*0.0528*	*0.0530*	*0.0537*	*0.0539*	*0.0543*	*0.0545*
Time trend, γ_10_	0.0106‡	0.0113‡	0.0097‡	0.0104‡	0.0100‡	0.0107‡	0.0091‡	0.0097‡
	*0.0012*	*0.0012*	*0.0009*	*0.0008*	*0.0012*	*0.0012*	*0.0008*	*0.0008*
Flu dummy, γ_20_	–0.0355‡	–0.0464‡	–0.0373‡	–0.0476‡	–0.0379‡	–0.0486‡	–0.0399‡	–0.0501‡
	*0.0053*	*0.0060*	*0.0050*	*0.0060*	*0.0054*	*0.0062*	*0.0051*	*0.0062*
Flu dummy × time trend, γ_30_	0.0002	–0.0005	0.0009	0.0002	–0.0002	–0.0009	0.0005	–0.0001
	*0.0009*	*0.0009*	*0.0006*	*0.0006*	*0.0009*	*0.0010*	*0.0006*	*0.0006*
No. observations	423	376	414	368	387	344	378	336
Hausman test statistic	<0.0001	<0.0001	<0.0001	<0.0001	<0.0001	<0.0001	<0.0001	<0.0001
	p>0.9999	p>0.9999	p>0.9999	p>0.9999	p>0.9999	p>0.9999	p>0.9999	p>0.9999
Breusch-Pagan test statistic	1525.90	1171.30	1535.75	1183.05	1403.42	1078.01	1422.01	1097.53
	p<0.0001	p<0.0001	pp<0.0001	p<0.0001	<0.0001	p<0.0001	p<0.0001	p<0.0001
Population change from Influenza, millions	–1.49	–2.02	–1.59	–2.05	–1.52	–1.98	–1.62	–2.01
Population change from Influenza, %	–2.72	–3.71	–3.03	–3.92	–3.23	–4.23	–3.62	–4.50
Population change, 1918 to 1919, millions	–0.75	–1.25	–0.96	–1.39	–0.93	–1.37	–1.14	–1.51
Population change, 1918 to 1919, %	–1.35	–2.26	–1.81	–2.63	–1.96	–2.88	–2.53	–3.34
Annual population growth rate to pandemic, %	1.06	1.13	0.97	1.04	1.00	1.07	0.91	0.97
Annual population growth rate after pandemic, %	1.08	1.08	1.06	1.06	0.98	0.98	0.96	0.96

*Italics indicate SE of the coefficient. †Chiba, Kanagawa, Shizuoka, Tokyo. ‡p<0.01.

**Figure:**
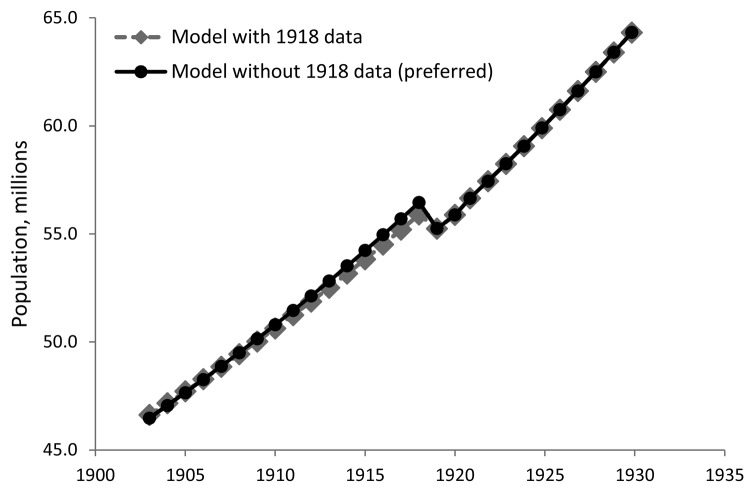
Effect of including 1918 data on estimated population of Japan. Data cover 1903–1930 and include observations for Hokkaido and the prefectures affected by the Kanto earthquake of 1923 (Chiba, Kanagawa, Shizuoka, and Tokyo).

Table 3 demonstrates that the models that control for other phenomena, including the Kanto earthquake of 1923, the 1898 and 1930 data, and the Hokkaido outlier, generate ranges of estimates that are similar to each other. Use of the alternative but less preferred A-type statistics (*6*) greatly increased the estimates of the number of deaths, thereby strengthening our conclusions. 

**Table 3:** Sensitivity analysis of influenza-induced change estimates for Japan

Controls for models	Ranges for estimated population change from influenza (millions)										
	1903–1930 data						1898–1935 data				
	Included			Excluded			Included			Excluded	
	Low	High		Low	High		Low	High		Low	High
Kanto earthquake prefectures*	–1.38	–2.02		–1.48	–2.17		–1.49	–2.05		–1.52	–2.01
Hokkaido outlier	–1.38	–2.12		–1.50	–2.17		–1.49	–2.02		–1.59	–2.05
1918 population count data	–1.38	–1.61		–1.97	–2.17		–1.49	–1.62		–1.98	–2.05
*Chiba, Kanagawa, Shizuoka, Tokyo.											

The only control that yielded distinct estimates conditional on its inclusion was the 1918 data control; the ranges of estimates for models that include the 1918 data (−1.38 to −1.61 million and −1.49 to −1.62 million) do not overlap with the ranges of estimates for models that exclude the 1918 data (−1.97 to −2.17 million and −1.98 to −2.05 million). Because other controls seem to have no material effect on the results, the final models selected are the ones in which the 1918 data are dropped but none of the other controls are implemented (i.e., the models in the second column of Tables 1 and 2). The estimated population loss is therefore 1.97 or 2.02 million persons, which translates to a drop in population of 3.62% or 3.71%.

## Discussion 

For nearly a century, Japan’s experience during the influenza pandemic of 1918–19 has been viewed as an anomaly within the broader Asian experience. In stark contrast with significantly higher estimates for deaths in Asia and globally, which themselves are often conservative, the standing mortality rates for Japan, based heavily on vital registration data, are <1%. There is, however, substantial reason to believe that vital registration data for the early 20th century in the most densely populated parts of Asia, including British India (*17*), the Dutch East Indies (*19*), and Japan (*8*,*20*), are inaccurate, suggesting the need for verification of mortality rates by using the Davis method (*17*), which is based on population count or census data. The key result of this study is that when these alternative population counts and census data are used, the experience of Japan conforms more closely to that of the rest of Asia; in Japan, rates of population loss approach 4% and an actual loss of ≈2 million. These estimates are similar to those for India (*17*,*22*). This result has implications for the large bodies of work on the epidemiology of the influenza pandemic of 1918–19 and, more broadly, the demographic history of Japan. Even adjusting for the possibility that a brief decline in fertility partly explains the population loss estimated in this study, the number of deaths in Japan were in all probability much higher than previously believed.

The results of this study come from using an alternative data source rather than vital registration data. Although the alternative data source is vulnerable to any inaccuracies inherent in the population counts and censuses of Japan, it nevertheless provides a way to confirm or contradict prior results that were based on vital registration data in the manner of Davis (*17*) and Chandra et al. (*22*) for India and Chandra (*21*) for Indonesia. Given the relatively reliable nature of population count and census data in comparison with vital registration data, however, the inaccuracies in the above analysis, in percentage terms, are probably smaller for population count and census data than for vital registration data.

A second possible limitation of the family of models estimated above is the implicit assumption of constant population growth rates for the periods before and after the pandemic. The analyses of Japanese demographers suggest some variation in birth and death rates during this period (*29*). Yet because we assumed stable population growth (derived from the differential between birth and death rates, with adjustment for migration), the models are tenable in view of the findings of these demographers of fairly stable population growth rates in Japan between 1900 and 1920 (*28*). The finding of population growth in the models (Tables 1, 2) that lies within the range of earlier estimates is further cause for confidence in these models.

Statistical by-products of this study include the substantial upward revision of the toll of the pandemic and the information about annual population estimates for Japan. The higher number of deaths should affect worldwide estimates of deaths from the pandemic published in studies, such as those by Patterson and Pyle (*2*) and Johnson and Mueller (*3*), and estimates about the epidemiologic characteristics of the disease in Japan that depend on those data. The annual population estimates for Japan should advance the rich literature for Japan as a whole and for the 47 prefectures by generating new estimates that explicitly account for the effect of the pandemic. Although the estimates for years distant from the influenza pandemic are similar to those produced by demographers, including Morita, Okazaki, and Yasukawa and Hirooka, they depart substantially from these estimates for 1915–1920, with implications for the earlier published works that have used these data.

Given the virulence of the influenza A(H1N1) virus that caused the disease and the continued worry caused by the possibility of its reemergence (*1*), this study dispels the myth that Japan was spared the ravages of the influenza pandemic of 1918–19. Japan is not an exception to be studied for possible solutions or measures that might ameliorate the effects of such an epidemic in the future. Rather, its experience is typical of that of other Asian countries for which we have more reliable estimates.

Technical Appendix Standard population growth model.
