# Supplementary material for: Deaths Associated with Influenza Pandemic of 1918–19, Japan
Source: Emerg Infect Dis. 2013 Apr;19(4):616–22. doi: 10.3201/eid1904.120103 (PMC3647405; doi:10.3201/eid1904.120103)
Supplement: Technical Appendix — Standard population growth model. [file 12-0103-Techapp-s1.pdf]

Article DOI: http://dx.doi.org//10.3201/eid1904.120103

Deaths Associated with Influenza Pandemic of 1918–19, Japan

Technical Appendix

Standard Population Growth Model

The model estimated in this paper is a standard population growth model with a breakpoint coinciding with the pandemic, adapted for panel data (i.e., multiple prefectures followed over multiple time periods) (*1,2*),

$$LPOP_{it} = \pi_{0i} + \pi_{1i}T_t + \pi_{2i}FLU_t + \pi_{3i}T_tFLU_t + \varepsilon_{it},$$

where $LPOP_{it}$ is the natural logarithm of population in prefecture i at time t; T_t is the linear time trend; FLU_t is the dummy variable representing the postpandemic years,

$$FLU_t = \begin{cases} 0, & t \leq 1918 \\ 1, & t > 1918 \end{cases}$$

ε_{it} is a random error term in the standard random coefficients model; and π_{0i}, π_{1i}, π_{2i}, and π_{3i} are random coefficients, each modeled as the sum of a fixed coefficient γ_{00}, γ_{10}, γ_{20}, or γ_{30} and a prefecture-specific random term with 0 mean. The random coefficients represent prefecture-specific estimates of the log of population in the initial year (1898 or 1903, depending on the model, π_{0i}), the rate of population growth before the pandemic (π_{1i}), the 1-time shift in the population trajectory due to the pandemic (π_{2i}), and the change in the rate of population growth after the pandemic (π_{3i}). SAS software was used to estimate the model (*3,4*). To compare various specifications of the model, we subjected them to 1) the Hausman test, used to determine whether a fixed effects specification is preferred to the random coefficients specification, and 2) the Breusch-Pagan test, which is used to determine whether the ordinary least squares specification is preferable to the random coefficients specification (Tables 1 and 2 in main article). Both tests favored the random coefficients specification.

References

1. Chandra S. Mortality from the influenza pandemic of 1918–19 in Indonesia. Popul Stud. 2013 Jan 23 [Epub ahead of print]. http://www.ncbi.nlm.nih.gov/pubmed/23339482

2. Chandra S, Kuljanin G, Wray J. Mortality from the influenza pandemic of 1918–19: the case of India. Demography. 2012;49:857–65. PubMed http://dx.doi.org/10.1007/s13524-012-0116-x

3. SAS Institute, Inc. SAS/ETS 9.2 user’s guide: the PANEL procedure. 2011 [cited 22 Dec 2011]. http://support.sas.com/documentation/cdl/en/etsug/60372/HTML/default/viewer.htm#panel_toc.htm

4. SAS Institute, Inc. SAS/STAT 9.2 user’s guide: the MIXED procedure. 2011 [cited 22 Dec 2011]. http://support.sas.com/documentation/cdl/en/statug/63033/HTML/default/viewer.htm#mixed_toc.htm
